# Therapeutic Potential of Human Intestinal Organoids in Tissue Repair Approaches in Inflammatory Bowel Diseases

**DOI:** 10.1093/ibd/izad044

**Published:** 2023-04-24

**Authors:** Duncan Rutherford, Gwo-Tzer Ho

**Affiliations:** Gut Research Unit, Centre for Inflammation Research, Queen’s Medical Research Institute, University of Edinburgh, Edinburgh, United Kingdom; Gut Research Unit, Centre for Inflammation Research, Queen’s Medical Research Institute, University of Edinburgh, Edinburgh, United Kingdom

**Keywords:** IBD, CD, UC, repair, organoids, stem cells

## Abstract

Inflammatory bowel diseases (IBDs) are chronic immune-mediated conditions characterized by significant gut tissue damage due to uncontrolled inflammation. Anti-inflammatory treatments have improved, but there are no current prorepair approaches. Organoids have developed into a powerful experimental platform to study mechanisms of human diseases. Here, we specifically focus on its role as a direct tissue repair modality in IBD. We discuss the scientific rationale for this, recent parallel advances in scientific technologies (CRISPR [clustered regularly interspaced short palindromic repeats]/Cas9 and metabolic programming), and in addition, the clinical IBD context in which this therapeutic approach is tractable. Finally, we review the translational roadmap for the application of organoids and the need for this as a novel direction in IBD.

**Key messages**

1. Novel restorative therapies are required in inflammatory bowel disease (IBD).
2. Organoid therapy is based on in vitro culture, followed by selection and expansion of healthy intestinal stem cells with a view to transplant into human intestinal mucosa.
3. Transplanted intestinal organoids can be applied to promote epithelial regeneration and restore normal intestinal physiology.
4. Key questions regarding organoid behavior on exposure to in vivo IBD conditions remain unanswered.
5. Further research is required to satisfy concerns regarding immunogenicity of allogenic and autologous organoid transplant as well as cumulative tumorigenesis risk.
6. Prorepair organoid therapy represents an important parallel approach to advanced immune-modulation drug treatment in defined clinical IBD settings.

## Introduction

The therapeutic approaches to Crohn’s disease (CD) and ulcerative colitis (UC), collectively the inflammatory bowel diseases (IBDs), are evolving rapidly. Since the first introduction of biologics, infliximab in 1999, multiple specific drug targets within the inflammatory pathways, via the use of monoclonal antibodies (eg, tumor necrosis factor α [TNFα], interleukin-23, interleukin-17) and, more recently, small molecules (eg, JAK inhibitors), have been developed.^1^ Notwithstanding these advances, there is a “therapeutic ceiling,” and despite the escalation of immune suppression, complete mucosal healing and steroid-free remission are difficult to achieve and are seen in <50% of patients.^2^ Such consistent observations emphasize the need for novel and complementary treatment strategies, particularly in those with extensive and severe disease features.

We previously reviewed the role for tissue repair approaches in IBD and posited a dual theoretical approach for complete mucosal healing encompassing parallel anti-inflammatory and proresolution/repair therapies.^3^ Many of the current drugs in the development pipeline or in clinical trials target inflammatory pathways and potentially modify cellular phenotype and composition of innate and adaptive immune cells within the inflamed IBD gut.^4^ Distinct from this, the last 10 years have seen dramatic progress in the development of organoid technology, the ability to grow and expand miniaturized organs in vitro, and the potential to use these organoids to repair tissue damage in many human diseases. Human intestinal organoids (HIOs) are now increasingly utilized to study relevant disease-related pathways/mechanisms and provide an improving platform to screen for drug targets with a strategic shift away from animal studies.^5^

In this review, we specifically focus on the translational application of adult stem cell (ASC)–derived HIOs as a defined tissue repair approach in IBD. We provide insights from related human diseases and remarkable progress seen in animal models, in particular, we discuss the future clinical context for its use. Finally, we illustrate how the creative synergistic recombination of HIO therapy with current technologies can lead to tangible progress from bench to clinic.

## The gut epithelium and the therapeutic rationale for HIOs in IBD

**Figure 1.**
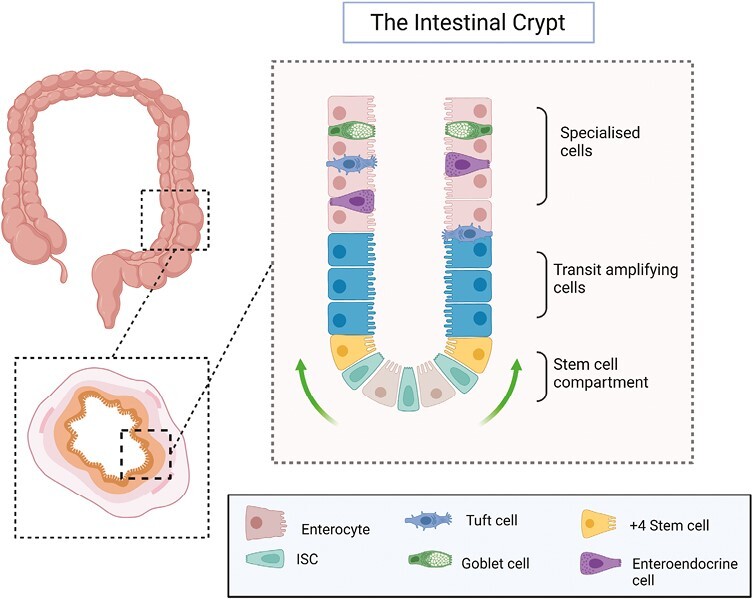
Structure of the intestinal epithelium. The intestinal epithelium is organized into units that consist of crypts and—in the small bowel—protrusions called villi. At the base of the intestinal crypt lies the stem cell compartment, where LGR5 intestinal stem cells (ISCs) divide and replicate, whereas +4 stem cells act as reserve stem cells. As ISCs divide, they advance along the crypt villus axis, first entering the transit-amplifying zone before differentiating into the specialized, terminally differentiated cells of the epithelium (eg, goblet, tuft, and enteroendocrine cells). Finally, at the tip cells undergo anoikis and shed into the lumen.

The gastrointestinal tract is lined by a single layer of columnar intestinal epithelial cells—a sophisticated multifaceted barrier that comprises several specialized cell types, each with a distinct function. Crucial to this lies in the ability of the gut epithelium to regenerate and renew in the face of a dynamic and hostile luminal environment. The constantly dividing intestinal stem cells (ISCs) that reside at the base of intestinal crypts continuously self-renew and proliferate replenishing specialized epithelial cells by differentiating and migrating toward the tip of the villus (Figure 1).^6,7^

**Figure 2.**
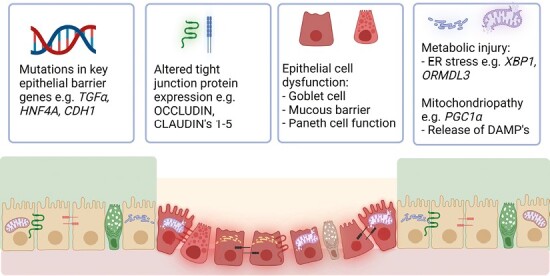
Overview of the causes of epithelial barrier dysfunction in inflammatory bowel disease (IBD). Genome-wide association studies and microarray studies of IBD patients have identified multiple risk loci in key epithelial genes (eg, *HNF4a*, *CDH1*, *REG4*). Furthermore, alterations in expression of key junctional proteins increase permeability and susceptibility to IBD. Specific alterations in function of epithelial cell subtypes are seen in IBD, for example a reduction in goblet cell abundance and the corresponding reduced mucus barrier layer seen in ulcerative colitis and Paneth cell aberrations in Crohn’s disease. Finally, metabolic and mitochondrial injury—of which DNA and mitochondrial encoding genes are frequently the most downregulated in active IBD—can lead to energy deficient states that reduce the epithelial barrier’s ability to regenerate after injury. DAMP, damage-associated molecular pattern; ER, endoplasmic reticulum.

In considering the rationale in the application of HIOs as a tissue repair approach in IBD, 2 factors are pertinent. Firstly, multiple lines of evidence point to a dysfunctional epithelium (innate and acquired) as a pathogenic factor leading to a breakdown in gut homeostasis and loss of barrier function in IBD (Figure 2).^7-9^ Secondly, specific damage to the ISCs results in further de-regulation and the loss of the capacity to regenerate a functional epithelium with a full complement differentiated cells.^10-12^ More importantly, there is accumulating evidence to show that ISC function is affected in IBD.^13,14^

In human CD, a reduction in the ISC population is observed within active disease compared with nonaffected gut mucosa.^15^ This is shown by correlating pathological assessment of activity with ISC frequency using in situ hybridization of the LGR5 stem cell marker. Similarly, in UC single-cell RNA sequencing (scRNA-seq) has provided in-depth analysis, confirming that ISC populations (via transcriptomic annotation) are reduced when comparing active with noninflamed epithelium.^16^ Here, the authors also found that noninflamed epithelium possessed a significantly enriched ISC population when compared with control samples. These lines of data suggest that perturbation of ISC function or potential may contribute to failure of IBD mucosa to heal or return to normal homeostasis. It is of interest that a low ISC population appears to predict future clinical recurrence in CD.^15^ On retrospective testing of noninflamed surgical resection margins, it was determined that crypts with both low levels of LGR5 expression and LGR5 expression in upper crypts were independently associated with ensuing disease recurrence.^15^ Thus, these early studies provide the premise for an intervention that is targeted toward the correction of ISCs in the IBD gut (either by replacement with healthy ISCs or, in the future, more precise scientific modification, augmentation, or restoration of ISC function, discussed in the following sections).

## From concept to development of HIOs

**Figure 3.**
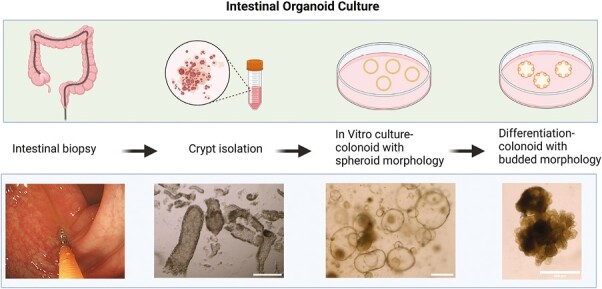
Human intestinal organoid culture. During routine endoscopy, 2 to 4 biopsies are sampled and stored on ice. Crypts are then liberated after incubation in EDTA solution for 60 minutes. During in vitro expansion, HIOs are maintained in a growth media that enforces a stem cell state. The expanded growth medium is modified by withdrawing growth factors and adding components that facilitate differentiation, as evidenced by increasing thickness, granularity, budding, and accumulation of intraluminal debris (scale bar = 250 µm).

Intestinal organoids, derived from adult ISCs are unique, self-organizing, multicellular, 3-dimensional cell culture systems that retain certain in vivo functions eg, secretion, absorption, contraction.^17-19^ Organoids can be cultured from isolated biopsies and expanded in vitro (Figure 3). Present techniques now allow differentiation into a full complement of cell subtypes representative of normal human tissue namely, stem cells, mature enterocytes, goblet, tuft, and enteroendocrine cells.^20-23^

**Figure 4.**
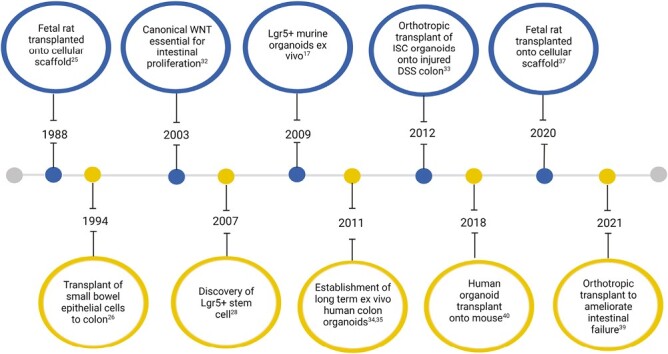
Timeline highlighting advances in organoid technology and transplantation. The first studies predated discovery of specialized media that drive intestinal stem cell (ISC) growth and expansion, instead digesting tissue whole and transplanting onto denuded animal intestine. Subsequent advances leveraged the discovery of the canonical WNT pathway and its influence on LGR5 stem cell in vitro culture. More recent studies have explored animal and later human intestinal organoids to heal damaged animal intestine and the restoration in normal physiology that this can generate. DSS, dextran sulfate sodium.

The tissue regeneration field, as a modern scientific discipline, came of age in the early 1990s (Figure 4).^24^ First, Vacanti et al^25^ showed that dissociated murine intestinal epithelium could be grown on polymer scaffolds and then subsequently transplanted in vivo into the omentum or mesentery of recipient animals. Despite its nonintestinal site of engraftment, transplanted cells developed villus-like structures with an epithelium populated with characteristic intestinal cell subtypes—suggesting an innate regenerative potential and property. Tait et al^26^ transplanted small bowel epithelial cells to denuded colonic mucosa in vivo, at 14 days the transplanted epithelium had engrafted and, of interest, had generated an epithelium with small bowel morphology. Despite the promise of these early pioneers, the field of transplantation failed to advance, limited by the inability to expand stem cell populations in vitro and the lack of knowledge in the mechanisms of gut tissue regeneration.^27^

A key discovery came with the discovery of the LGR5 marker for stem cells of the intestine and colon.^28^ Subsequently, Sato et al^17^ successfully demonstrated that the addition of key growth factors^29-32^ permitted in vitro culture of LGR5 stem cells from intestinal crypts, thus establishing the initial platform for organoid culture. The seminal paper by Yui et al^33^ displayed successful differentiation of murine colonic ISCs in vitro, demonstrating microvilli development and formation of junctional complexes within the cultured enterocytes. The authors subsequently performed orthotropic organoid transplant via the expanded EGFP-labeled stem cell population onto dextran sulfate sodium (DSS)–mediated colitis. Several key findings are noteworthy. Firstly, the transplanted HIOs engrafted growing into self-renewing crypts were histologically indistinguishable from native epithelium. Secondly, there was a functional physiological improvement in the recipient mice compared with those that did not receive the organoids. Thirdly, transplanted organoids engrafted in areas with colonic mucosal damage. These collective data point to an early potential for organoids as a tissue repair approach in humans.

Following this breakthrough, further key questions were resolved, namely (1) whether ISCs can maintained in in vitro culture long term^34,35^; (2) whether transplanted ISCs can differentiate into relevant and functional competent enterocytes that respond to systemic signaling^36^; and (3) if ISCs can be successfully expanded into sufficient quantities for translational purposes.^37^ However, the most immediate translational step concerns the bridging the predominantly mouse work into humans.

## HIOs in IBD: From mouse to humans

While there is exciting progress in this field, there are realistic limitations to progress toward human translation. To date, all but 1 trial modeling organoid transplant^38^ has used immunodeficient mouse recipients,^33,36,37,39-42^ and clearly, further evaluation in hosts with more complex immune systems is required. Also, the predominantly used DSS-colitis model in these studies is more akin to an injury model that does not recapitulate the complex multifactorial immune-mediated human IBD pathogenesis.^33,41,43^ Notwithstanding these caveats, in the mouse, scRNA-seq analysis of colonic epithelium after orthotopic transplantation has shown that transplanted regions regained a similar cellular composition to normal healthy epithelium, maintaining similar populations of goblet, Paneth, enteroendocrine, and tuft cells, and enterocytes.^44^ Hence, there is confidence that a full complement of enterocytes, rather than a selected population, can arise following organoid transplantation in mouse.^45^ However, in human IBD scRNA-seq studies, there are clear differences in epithelial cell populations seen in both CD and UC with alterations in proportions of epithelial, goblet, tuft, enteroendocrine, and M cells as well as proportions of colonocytes.^16,46,47^ It is not yet known if HIO transplantation into IBD gut can similarly restore normal enterocyte population.

Furthermore, clinical pertinent scientific questions also become obvious as more human-focused primary organoid work continues to emerge in human IBD. Primarily, what is the effect of environmental cues on the characteristics of ISCs and HIOs, especially inflammation? More fundamentally, are IBD ISCs inherently programmed to retain proinflammatory properties that predispose to IBD in the first place?

Initial studies displayed that HIOs generated from inflamed gut quickly (~2 weeks) lose their inflammatory transcriptomic profiles and phenotype during in vitro culture, as evidenced in more traditional bulk RNA sequencing data.^48,49^ Instead, IBD-derived HIOs (from both inflamed and uninflamed tissues) coalesce, maintaining distinct transcriptomic and epigenetic phenotypes to that of control populations.^45,49,50^ Specifically, there has been a reported 90% concordance between differently expressed genes in tissue and in tissue-derived organoids in CD.^49^ These studies suggest that the presence of inflammation does not translate to a unique and imprinted proinflammatory IBD HIO transcriptomic pattern in cultured conditions. Following this, Arnauts et al^48^ showed that reintroduction of the inflammatory environment with a cocktail of TNFα, interleukin-1β, and flagellin resulted in a higher proinflammatory gene expression response in UC than non-IBD HIOs. This suggests HIOs from IBD may display the same proinflammatory potential upon re-entry into an inflammatory environment such as the active IBD gut milieu; however, the mechanisms for this are unclear.

Alongside the reduced ISC populations observed in IBD,^15^ the impaired capacity of IBD-derived organoids to grow in vitro^51,52^ further contributes to the notion that ISCs in IBD are dysfunctional.^53^ In the mouse gut, a recent study identified 3 distinct stem cell populations based on scRNA-seq, indicating that ISC biology may be more complex.^54^ Type I slow cycling stem cells are enriched in LGR5, possessing more stem-like features, whereas type II and III ISCs are more differentiated and proliferative, concurrently expressing major histocompatibility class II. The balance of these distinct populations is important. Biton et al^54^ showed that upon exposure to inflammation (here, *Salmonella* infection was used), there is a suppression of type I ISCs with a shift to type II + III ISCs, toward the nonconventional antigen-presenting cell type. Kanke et al^14^ subsequently confirmed this finding in human CD, whereby nonactive areas were found to possess not only a reduction in overall ISC number, but also a significant downregulation of ISC type I, and upregulation of type II + III ISC subtypes. Finally, in CD organoids, inflammatory stimulation with interferon γ led to a upregulation of major histocompatibility class II expression when analyzed through both immunohistochemistry and bulk RNA sequencing.^55^ Thus, it is conceivable that in IBD the ISCs are fundamentally different, in that not only are they reduced in number, but also those that are present are not suited to assist in repair and regeneration of the epithelium. Whether this is a precursor or in response to previously undetected inflammation remains unclear.

## Toward clinical application: Lessons from relevant human diseases

Synergistic advances in relevant scientific fields provide new directions for research toward HIO clinical translation—namely CRISPR (clustered regularly interspaced short palindromic repeats) for gene editing, metabolic programming, and the development of a cogent approach linking mouse and human data in the development of an apposite ex vivo model in tissue regeneration. These are at an early stage in development, and we provide an overview of their potential relevance. In simple terms, these scientific tools may make the idea of correcting specific defects and replacing ISCs possible, in very defined clinical situations in IBD (to be discussed in the following sections).

### CRISPR/Cas9 gene editing

In cystic fibrosis, the first successful CRISPR-based gene correction was reported in gut organoids from cystic fibrosis patients. Here, the CFTR locus of affected organoids was modified by insertion of a normal *CFTR* gene using CRISPR/Cas9, with targeted organoids displaying a restored swelling response to forskolin induced swelling assay.^56^ In colorectal cancer, CRISPR/Cas9 is exploited to develop a closer human colorectal cancer model using HIOs. Roper et al^58^ introduced deletion of *APC* and *p53* tumor suppressor genes into murine organoids that were subsequently orthotropically transplanted into murine colon,^57^ providing a faster approach over traditional colorectal cancer models. Both examples provide a scientific opportunity to modify the IBD ISC genetic susceptibility (for example, in *NOD2* mutations in Paneth cell dysfunction in CD)^59^ and to develop a human IBD epithelial experimental model with the ability to perturb and interrogate function with gene editing.^60^

### Metabolic programming

Because mitochondrial dysfunction is a notable pathogenic component in ISCs and IBD,^61^ and with recent strides in metabolic programming in epigenetic imprinting of cellular function,^62^ 2 recent studies provide insights into how correction of mitochondrial-metabolic factors in HIOs resulted in improvement in ISC function. Jackson et al^63^ showed that deletion of prohibitin-1 (*Phb1*), a gene encoding a major component protein of the inner mitochondrial membrane protein, caused mitochondrial dysfunction and clinical spontaneous murine ileitis. They then subsequently showed that mitochondrial antioxidant therapy^64^ prevented *Phb1* deletion–mediated ileitis with parallel data to show a positive effect on intestinal organoid growth from *Phb1*^iΔiec^ mice. Similarly, another study focusing on metabolic reprogramming in IBD observed that crypts from inflamed regions of TNF^ΔARE^ mice fail to grow into organoids.^15^ The authors then reinforced mitochondrial respiration through addition of dichloroacetate (DCA) to organoid growth media. DCA, a Food and Drug Administration–approved drug, acts to shift adenosine triphosphate generation from glycolysis to oxidative phosphorylation through targeting of pyruvate dehydrogensase.^65^ Resultantly, DCA addition facilitated successful organoid culture from inflamed regions. Remarkably, after 8 days of treatment, removal of DCA did not lead to a reversal in the phenotype or morphology of organoids from inflamed regions as compared with wild-type mice, suggesting that these effects could be long lasting. These lines of data open the possibility to reprogram metabolically defective ISCs and subsequent downstream epithelial phenotypes using HIOs.

### Tissue engineering and repair

Driven by a major unmet clinical need, organoid technology has received much attention, particularly in the field of bile duct surgery in which supply of donor tissue is a major limiting factor for reparative surgery for bile duct disorders.^66^ In this context, 2 elegant sequential studies by Sampaziotis et al^67,68^ combined human and mouse work with subsequent creative development of an ex vivo perfusion model to demonstrate how cholangiocyte organoids can be used to regenerate bile ducts. First, they showed that human cholangiocyte organoids impregnated on polyglycolic scaffolds can facilitate the healing gallbladder incisions and replace sections of the common bile duct in mouse.^67^ These recipient mice survived for over 1 month, retaining normal liver function, and the engineered epithelium was able to self-renew, maintaining a patent bile duct lumen. In a follow-up study, they also demonstrated that intraductal organoid infusion of gallbladder cholangiocytes can heal induced cholangiopathy.^68^ Building on the success of murine models, Sampaziotis et al have subsequently bridged the gap toward human therapy by using an ex vivo organ perfusion model. Here, they transplanted labeled cholangiocytes into the ducts of normothermic perfusion circuit liver grafts taken from deceased human donor’s with ischemic duct injury.^68^ Transplanted organoids successfully grafted and regenerated 40% to 85% of intrahepatic bile ducts. Intervention recipients showed evidence of ultrasonographic healing of bile ducts, while control recipients showed ischemic injury and loss of epithelial continuity. The significance of these findings is potentially major, with direct relevance to liver transplantation and bile duct surgery, paving the way for the first human trials.

These 3 examples of scientific technological advances, while disparate in nature, provide directions to the next translational steps of HIO technology in IBD. Notwithstanding the necessary progress still required at this early stage, we discuss the clinical context for the use of HIOs in IBD in the following section.

## Clinical context for therapeutic application HIOs in IBD

In terms of realistic clinical translation, the approach taken by local application of mesenchymal stem cell therapy for perianal fistulas provides an roadmap for translation of HIOs into the clinic.^69^ Therapeutics in this field have been through multiple trial phases culminating in the Adipose derived mesenchymal stem cells for induction of remission in perianal fistulizing Crohn’s disease study, a phase 3 randomized controlled trial involving 212 patients.^70^ Although this treatment has not gained wide clinical acceptance, in lieu of cost and limited long-term efficacy, the concept of transitioning a stem cell–based treatment from the bench to bedside and scaling of trials for regulatory approval provides promise to replicating the same for HIO therapy.^71^

**Figure 5.**
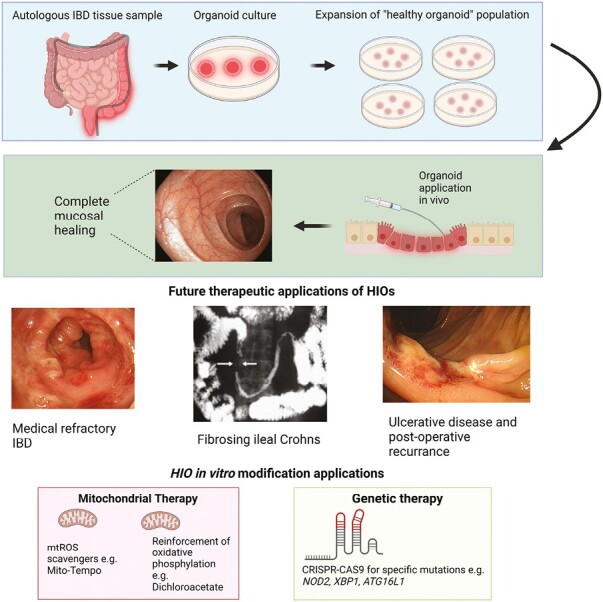
Proposed application of human intestinal organoid (HIO) therapy. First, HIOs are isolated from patient via endoscopic sampling for subsequent autologous application. HIOs are expanded in vitro to generate a healthy pool of organoids. Once sufficient quantities have been achieved, HIOs are transplanted endoscopically. Specific clinical situations for which HIO application may be utilized include restoration of epithelial barrier as rescue therapy for medically refractory inflammatory bowel disease (IBD), fibrosing Crohn’s disease phenotypes, and early postoperative recurrence. Future in vitro culture therapy may include correction of aberrant metabolic phenotypes with mitochondrial therapy or targeted genetic therapy for specific pathogenic mutations. CRISPR, clustered regularly interspaced short palindromic repeats; mtROS, mitochondrial reactive oxygen species.

How widely applicable HIOs are in the real-world clinic is unclear, given the present early stage of research. We envisage several hypothetical clinical scenarios in which HIOs might be relevant (Figure 5): (1) in medically refractory IBD with significant gut damage; (2) in early postoperative recurrence of CD with localized inflammation in the operative anastomosis; (3) in fibrostenosing CD, in which animal studies have provided some data to suggest benefit; (4) in targeting of specific defects with known functional implications (eg, *NOD2* gene variants with associated Paneth cell dysfunction, mitochondrial bioenergetics defects in UC)^15,63,72^; and (5) in the increasing prevalent advanced-age IBD in which the impaired ageing gut barrier function is more relevant.^73,74^

In studies investigating organoid therapy to restore the epithelial barrier function, Sugimoto et al^40^ used EDTA and a mechanical treatment to remove areas of epithelium of colonic murine colon. Onto these areas they subsequently transplanted GFP-labeled human colonic organoids with overall engraftments of 75% with similar rates being found in other studies.^75^ The engrafted organoids successfully healed areas of epithelium and retained a human phenotype both in terms of shape and size of villi, but also functionally, as delineated by AB^+^ and PAS^+^ goblet cell distribution within crypts. Jee et al^44^ also found that orthotropic engraftment was able to restore epithelial barrier integrity, as tested by exposure to TRITC-dextran. In a model more representative of IBD, after exposure to DSS colitis, organoid transplant recipient mice were found to have areas of healed mucosa, with a full complement of differentiated cell types.^33^ Moreover, transplantation was associated with a significant increase in body weight when compared with sham.

HIOs could potentially be applicable in fibrostenosing CD. Jee et al^44^ modeled orthotropic organoid transplantation to heal damage induced by radiation exposure. Organoids were transplanted at 6 and 10 days after radiation exposure (50 Gy). In addition to successful regeneration of murine mucosa, there was also a significant reduction in collagen accumulation and reduction in submucosal thickness, suggesting that HIOs can also ameliorate postinjury fibrosis.

## ASC-derived vs pluripotent stem cell organoids

While our review has focused on ASC HIOs with its clearer translational pathway, pluripotent stem cells (PSCs) derived from embryonic tissue or reprogrammed somatic cells (induced pluripotent) offer an alternative approach. Here, PSCs can be differentiated into nearly any cell type via differentiation protocols that recreate embryological pathways.^76^ The differences of ASC HIOs vs PSC HIOs are summarized in Table 1. Unlike ASC HIOs, when intestinal PSCs are cultured in a 3-dimensional environment, they generate more complex models that contain both mesenchymal and epithelial tissue.^36,77^ This more complex composition may have theoretical implications, in that they are better suited to mucosal healing ambitions with the additional involvement of the mesenchyme offered by PSC HIOs. In fact, this approach has been tested successfully in murine colonic injury models, although its efficiency has not be directly compared with that of ASC HIO transplantation.^41,42^

**Table 1.** Key differences between intestinal adult stem cell and pluripotent HIOs.

	Adult stem cell HIO	PSC HIO
Source, with common examples	• Endoscopic mucosal biopsy, surgical resection	• iPSC (any somatic cell) (eg, blood, adipose tissue) • Embryonic tissue
Advantages	• Stable genetic signature • Rapid establishment and expansion • Long-term in vitro culture achievable	• Differentiation into multicell lineages (e)g, mesoderm and endoderm
Disadvantages	• Fixed differentiation lineages	• Fetal signatures in PSCs • Risk of unwanted cell types and tumorigenesis • Complex, lengthy protocols to generate HIOs • Challenging to maintain in vitro long term

Abbreviations: HIO, human intestinal organoid; iPSC, induced pluripotent stem cell; PSC, pluripotent stem cell.

However, this added multilineage cell complexity also presents formidable hurdles to clinical translation. For example, there is greater risk of genomic instability and thus development of undesirable cell type development (or even teratoma risk).^78^ Notwithstanding this, significant progress in PSC-derived organoids is evident and highly relevant as a human intestinal experimental platform to study the development and homeostatic processes of the gut.^79^

## Barriers to translation of HIO transplantation in IBD

There remain many formidable practical considerations in the translational development (Box 1). Relevant to ASC-derived HIOs, generation of large batch “healthy” organoids for clinical use will introduce new challenges in scaling, standardization, and automation of production. The method of application (ie, direct endoscopic injection), how to perform first-in-human testing, and in what IBD subgroup this will be relevant are key questions. Importantly, there is a question surrounding the potential immunogenicity of transplant medium. Currently the most utilized substance in experimental models is a commercially available substance called Matrigel (Corning).^80,81^ With its undefined composition derived from Engelbreth-Holm-Swarm mouse sarcoma and issues with batch-to-batch variability, licensing for medical use in humans would be challenging.^82,83^ Here, PSC-derived HIOs that are differentiated under xenogeneic-free conditions offer an advantage. Some research groups have transitioned transplant medium toward using substances such as fibrin, which is already licensed for human use,^44,84^ and hydrogel, which can be readily genetically altered and can adapted to mimic the gastrointestinal microenvironment.^83,85^ It remains unclear whether use of 3-dimensional growth matrices during the in vitro expansion phase of organoid culture has any carryover immunogenic effect upon transplant.

**Box 1: Key issues for clinical translation of human intestinal organoids in inflammatory bowel disease**

**Immunogenicity—will human intestinal organoids induce immune response?**

1. Allogenic pluripotent stem cells/mesenchymal stem cells do not retain full human leukocyte antigen immune evasiveness.^87–91^
2. Allogenic mesenchymal stem cells have good safety profiles for patients with inflammatory bowel disease at up to 1 year.^70,92–95^
3. Current animal models for organoid transplantation have used immunocompromised recipients.^33,36,37,39-42^
4. It is unclear if intestinal derived adult stems will benefit from the same partial immune privilege seen in pluripotent stem cells/mesenchymal stem cells.
5. Autologous human adult stem cell transplantation would minimize immunogenicity but is expensive and would prevent “off-the-shelf therapy.”
6. It is unclear if human intestinal organoid culture components will increase risk of transplant reactions (eg, 3-dimensional growth matrix).

**Neoplastic potential**

1. In vitro intestinal stem cell culture may promote point mutations.^96^
2. It is unclear whether point mutations will persist or cells enter senescence upon transplantation.^97^
3. Long-term (6-month) organoid culture has not detected karyotype alterations.^33,41^
4. A large systematic review of mesenchymal stem cell therapies (62 randomized controlled trials with 3546 patients, follow-up of 6 months to 2 years) found that there were no reports of tumorigenesis.^98^

## Conclusions

Our review presents the conceptual opportunity and vision of using HIOs as a tissue repair approach in IBD. Organoid technology is a perceptible advance in translational science and the study of human diseases. From a human experimental model perspective, it is salutary to note that this is unlikely to capture the complexities of a multifaceted immune-mediated condition such as IBD, and some realistic appraisal is needed. From a tissue repair approach, different but equally formidable challenges into how this can be applied in the clinic are noteworthy. Recent studies in bile duct repair provide a template for how HIOs can be further developed and applied in IBD. Furthermore, mesenchymal stem cell therapy in perianal CD (now in the clinical space) provides a roadmap for the translational process.^86^ Presently, HIO tissue repair approach is mostly likely to be relevant in a defined IBD group. While challenges remain, highly exciting progress that is driven by the clinical unmet need of repairing tissue damage in the IBD gut and many other human diseases provides optimism for future clinical application of this novel approach.
